# Clinical analysis of Hem-o-lok closure of the left subclavian artery stump in acute Stanford type A aortic dissection

**DOI:** 10.3389/fcvm.2024.1472815

**Published:** 2024-11-21

**Authors:** Jian-Qiang Li, Tu-Min Sha, Ping Dong, Xiao-Xia Li, Peng Zhang, Zhen-Qing Zhao, Lin-lin Jie, Lei Zha, Chao-Liang Liu

**Affiliations:** ^1^ Department of Cardiac Surgery, Yantai Yuhuangding Hospital, Yantai, China; ^2^ Department of Hematology, Yantai Yuhuangding Hospital, Yantai, China; ^3^ Department of Pathology, Yantai Yuhuangding Hospital, Yantai, China; ^4^ Department of Anatomy, Shandong College of Traditional Chinese Medicine, Yantai, China

**Keywords:** Hem-o-lok, acute Stanford type A aortic dissection, cardiovascular, left subclavian artery (LSA), surgical operation

## Abstract

**Objective:**

This study aims to summarize the clinical experience of using Hem-o-lok clips for the closure of the left subclavian artery (LSA) stump in patients with acute Stanford type A aortic dissection.

**Methods:**

Clinical data were collected from 96 patients with acute type A aortic dissection admitted to our hospital from January 2020 to December 2022. The cohort comprised 61 males and 35 females, with an average age of 52 ± 9.57 years and a mean body weight of 79.54 ± 12.57 kg. The mean diameter of the LSA opening was 11.24 ± 1.48 mm, as measured by computer tomography angiography (CTA) of the thoracoabdominal aorta. All patients underwent emergency Sun's procedure. The surgical method for the LSA stump was selected based on the anatomical location, depth of the subclavian artery, vessel diameter, length of the free vessel, and extent of dissection. Techniques included the use of two Hem-o-lok clips, 5–0 prolene suture with one Hem-o-lok clip, and 5–0 prolene suture alone.

**Results:**

All the patients successfully completed Sun's procedure as planned. Two Hem-o-lok clips were used to close the stump of the LSA in 38 cases. A combination of 5–0 prolene suture and one Hem-o-lok clip were used to close the stump of the LSA in 54 cases. The LSA stumps for 4 cases were closed with 5–0 prolene suture only. Postoperative complications included cerebral infarction in one patient, renal insufficiency in one patient, and gastrointestinal bleeding in one patient. There were no surgery-related deaths, no paraplegia,and all patients were successfully discharged. The 1-year follow-up CTA of the thoracoabdominal aorta demonstrated effective thrombosis of the false lumen in the stented segment of the thoracic aorta, no aneurysmal dilatation, and successful closure of the LSA stump.

**Conclusions:**

The simplified approach of Hem-o-lok closure of the LSA stump largely mitigates the difficulty in the LSA operation process,shortens the time of operation and reduces bleeding risk, thus effectively improving a patient's prognosis and yielding satisfactory clinical outcomes.

## Introduction

Acute Stanford type A aortic dissection (AD) represents a critical cardiovascular emergency, also known as “super cancer”. Mortality increases by approximately 1% for every hour delay post-onset (1). Computed tomographic angiography (CTA) of the thoracoabdominal aorta has become the preferred method for the diagnosis of AD due to its advantages of simplicity, speed, safety, reliability, high sensitivity and specificity. It can provide sufficient information for the diagnosis and treatment of AD, so as to facilitate adequate preoperative preparation and smooth and accurate intraoperative operation (2). Surgical intervention is the main treatment modality to improve patient outcomes (3). Currently, Sun's procedure, which involves total aortic arch replacement combined with an elephant trunk stent, is the standard surgical approach for treating acute type A aortic dissection (4). During Sun's procedure, addressing the branches of the aortic arch, particularly the left subclavian artery (LSA), poses significant challenges. The LSA is anatomically difficult to expose and anastomose because of its deep position and the displacement of the three branches of the aortic arch to the left posterior, exacerbated by the enlarged false lumen of the dissection (5). Therefore, ensuring safe and effective closure of LSA stumps remains a major challenge for vascular surgeons during the reconstruction of the aortic arch in acute type A dissection. Traditional methods for treating LSA stumps involve silk ligation or sliding suture closure. These techniques are demanding due to the deep anatomical location and unhealthy adventitia of the aorta. Any bleeding during these procedures can be extremely difficult to control and may lead to catastrophic outcomes. To address these challenges, our center has adopted the Hem-o-lok vascular clips for LSA stump closure. This technique simplifies the surgical process, reduces operational difficulty, and minimizes the risk of bleeding.

## Materials and methods

### Patients

This study retrospectively analyzed the clinical data of 96 patients with acute type A aortic dissection admitted to our center from January 2020 to December 2022. The cohort consisted of 61 males and 35 females, with a mean age of 52 ± 9.57 years and a mean weight of 79.54 ± 12.57 kg. Among these patients, 57 had dissection involvement of the LSA ostium, while 39 did not. Preoperative echocardiography revealed aortic valve closure in 75 cases and hemopericardium effusion in 62 cases. Based on the spatial configuration information of aortic arch provided by CTA of the thoracoabdominal aorta and the anatomical location, depth, vessel diameter, vessel free length and anatomic involvement degree of the LSA, the surgical plan was formulated. The diameter of the LSA was measured with an average of 11.24 ± 1.48 mm (Table 1).

**Table 1:** Patients’ data [cases (%)/(x¯ ± s)].

Patients	Data
Age (years)	52 ± 9.57
Weight (kg)	79.54 ± 12.57
Male (cases)	61 (64%)
Female (cases)	35 (36%)
LSA ostia involved by dissection (cases)	57 (59%)
Without LSA ostia involved by dissection (cases)	39 (41%)
Diameter of LSA (mm)	11.24 ± 1.48
AI (cases)	75 (78%)
Pericardial effusion or hemorrhage (cases)	62 (65%)

### Surgical management

The surgery was performed under general anesthesia with extracorporeal circulation. A midline thoracotomy was performed to access the aortic arch, with cannulation of the right axillary cavity and/or femoral artery, and right venous return was established for extracorporeal circulation. Cooling was initiated, the ascending aorta was clamped, and the HTK myocardial protection solution was perfused around the coronary artery branches. Aortic valve reconstruction was performed using the leaflet suspension method, Bentall procedure, and David procedure. The sinotubular junction was reinforced using the sandwich method, adventitial inversion technique, and pericardial strip lining. Root reconstruction involved blind anastomosis.

Once the rectal temperature dropped to 28℃, the three major branches of the aortic arch were clamped and lower body circulatory arrest with bilateral cerebral perfusion was initiated. The left common carotid artery and LSA were carefully isolated from surrounding tissues. The aorta was clamped between the left common carotid artery and LSAs based on anatomical location, depth, blood vessel diameter, free length of blood vessels, and dissection involvement. The LSA stump was managed using two Hem-o-lok vascular clips, one Hem-o-lok vessel clip plus 5–0 prolene suture, and continuous 5–0 prolene suture for stump closure. Appropriate artificial vascular stents were placed in the descending aorta, ensuring proper alignment with the aortic arch. A four-branch artificial graft was selected, and the distal anastomosis was completed with a continuous 4–0 prolene suture, restoring lower body perfusion. The four-branch graft was sequentially anastomosed to the LSA, left common carotid artery, and anonymous artery. Proximal aortic anastomosis was performed concurrently, completing the reconstruction of the ascending aorta and aortic arch.

Postoperatively, all patients underwent CTA of the thoracoabdominal aorta before discharge. Follow-up was conducted via telephone, WeChat, and outpatient visits at 1, 3, 6 and 12 month(s) post-discharge, with repeat CTA of the thoracoabdominal aorta at each follow-up interval.

### Statistical analysis

The data were analyzed using SPSS statistical software. The measurement data were expressed as mean ± standard deviation (x¯ ± s). Data with a normal distribution were analyzed using *t*-tests and variance analysis, with a significance level set at *P* < 0.05.

## Results

**Figure 1:**
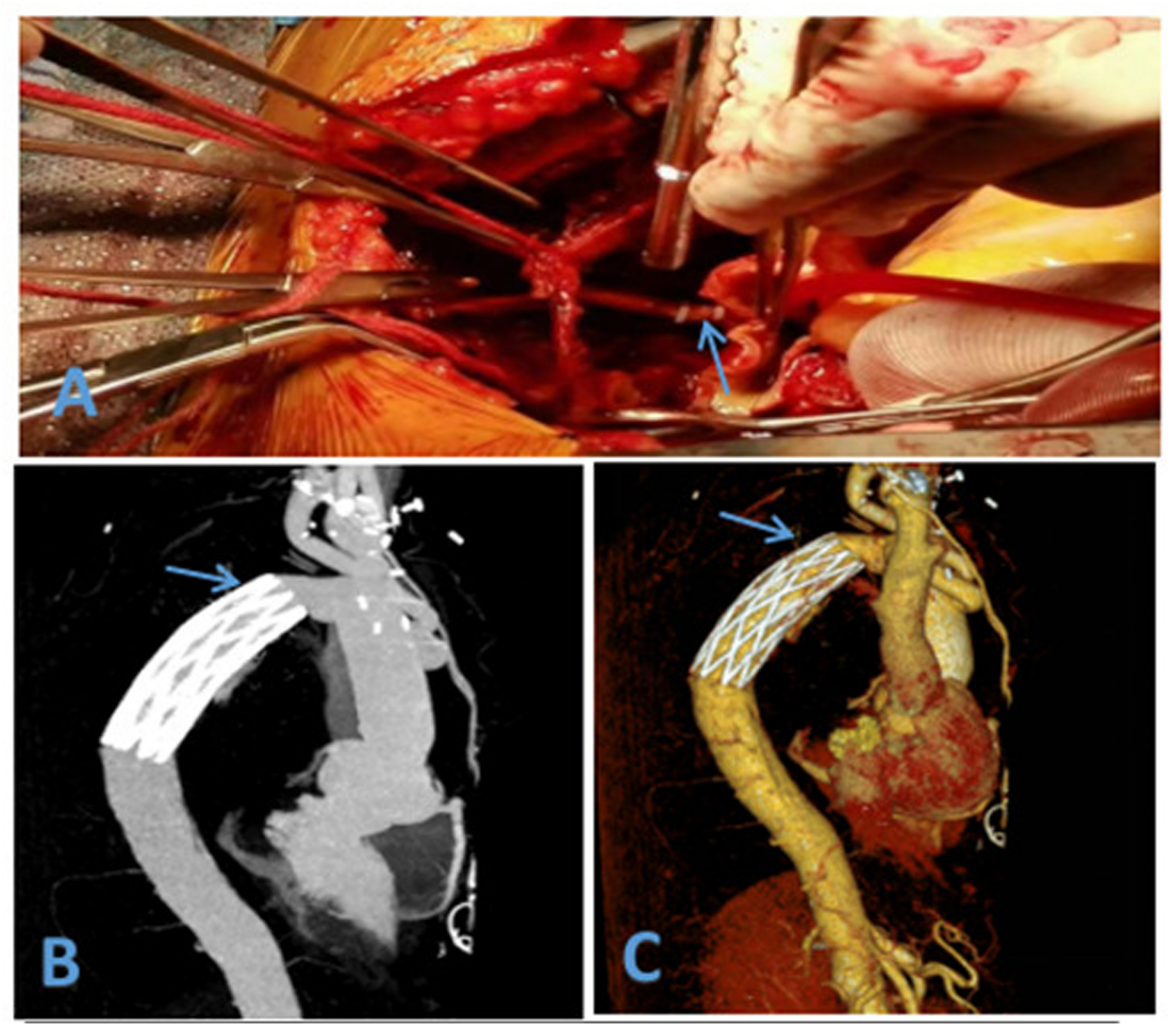
**(A)** Hem-o-lok vascular clip used to close the LSA **(B)** postoperative CTA **(C)** postoperative CTA (3D).

**Figure 2:**
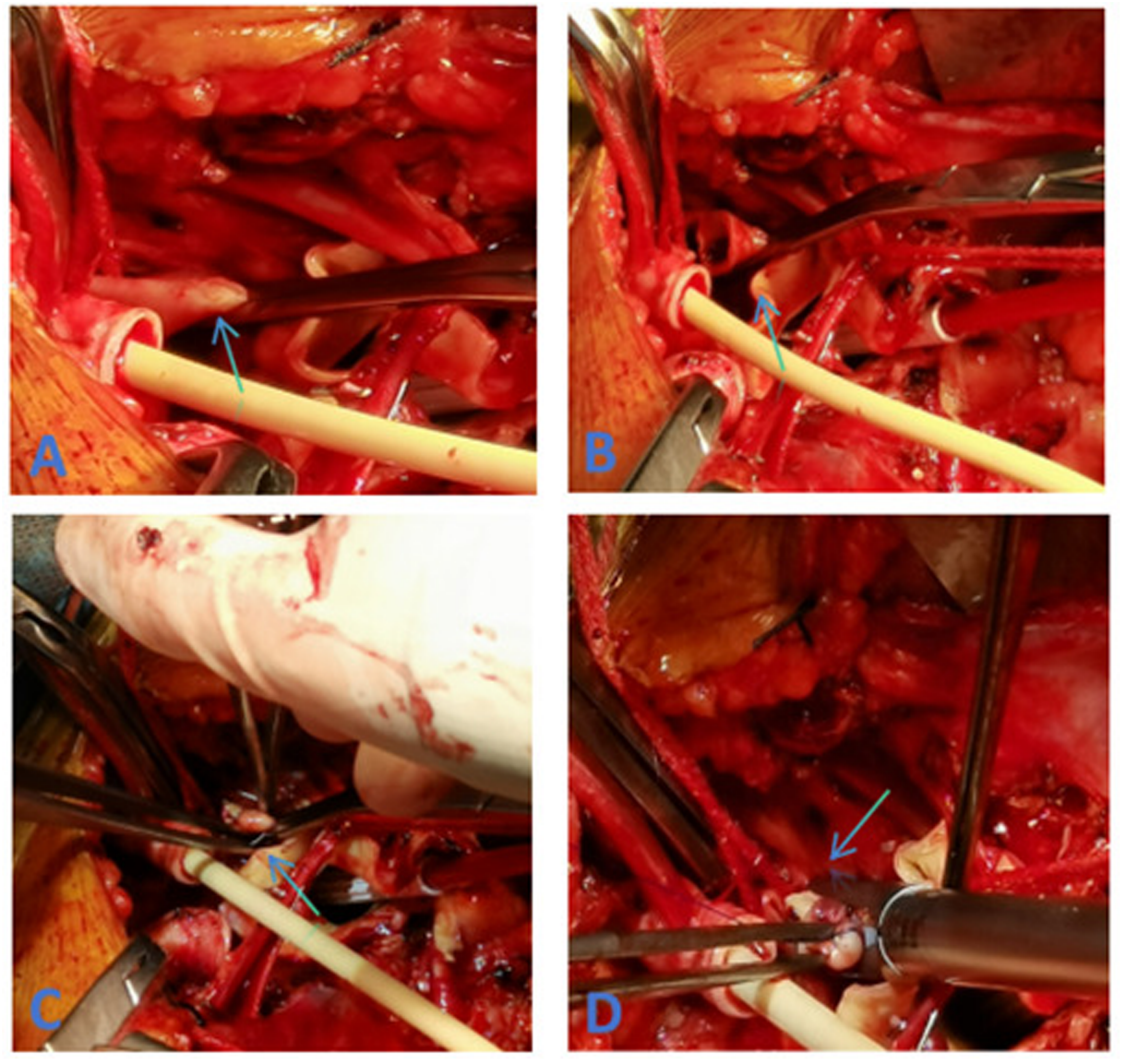
**(A)** Clamping of the proximal LSA **(C)** closure of the LSA stump using 5–0 prolene sutures **(B)** resection of the LSA **(D)** application of Hem-o-lok vascular clips to secure the LSA stump.

All patients successfully underwent Sun's operation as planned. The aortic valve was managed using Bentall and David procedures, with the sinus-tube junction using the sandwich method, outer membrane varus method, and blind anastomosis. The lenght of endovascular prosthesis is selected according to the height and weight of the patient, including 100 mm (35 cases) and 120 mm (61 cases) (Table 2). For the LSA stump, 38 cases were treated with two Hem-o-lok vascular clips (Figure 1), 54 cases with one Hem-o-lok clip and continuous 5–0 prolene suture (Figure 2), and 4 cases with continuous 5–0 prolene suture alone. The operative time was 406.64 ± 92.45 min, Cardiopulmonary bypass(CPB) time was 167.52 ± 44.68 min, aorta cross-clamp time was 103.73 ± 46.64 min, and hypothermic circulatory arrest(HCA) time 28.64 ± 10.11 min (Table 2).

**Table 2:** Surgical data [cases (%)/(x¯ ± s)].

Surgical data	Data
Time of Operation (min)	406.64 ± 92.45
Time of CPB (min)	167.52 ± 44.68
Time of Aortic Cross-Clamp (min)	103.73 ± 46.64
Time of HCA (min)	28.64 ± 10.11
Preoperative Bleeding (ml)	1194.71 ± 491.95
Treatment method of LSA Stump and diameter of LSA	
(a) 2 Hem-o-lok clips	38 (39.6%)
Diameter of LSA (mm)	10.98 ± 1.24
(b) 2 Hem-o-lok clips + 5–0 prolene suture	54 (56.2%)
Diameter of LSA (mm)	11.63 ± 1.66
(c) 5–0 prolene suture only	4 (4.2%)
Diameter of LSA (mm)	14.64 ± 0.71
Lenght of the endovascular prosthesis	
110 mm (cases)	35 (36.46%)
120 mm (cases)	61 (63.54%)

Postoperatively, all patients were transferred to the intensive care unit for continuous treatment. The mean duration of ventilator support was 40.95 ± 16.41 h, and the average hospital stay was 17.83 ± 5.27 days. There were one case of cerebral infarction, one case of renal failure with preoperative long-term hemodialysis which hospital stay was 23 days, and one case of gastrointestinal bleeding; all other patients recovered without complications. There were no surgery-related deaths, no paraplegia,and all patients were successfully discharged. Pre-discharge enhanced CTA of the thoracoabdominal aortic showed no aneurysmal expansion in the stented segment, and the LSA stumps were well closed (Figure 1). All patients were followed up at 1, 3, 6, and 12 month(s) post-discharge with repeat CTA of the thoracoabdominal aortic. During the 1-year follow-up period, there was good thrombosis in the false lumen, no aneurysmal expansion, and well-closed LSA stumps. There were no aorta-related deaths, and satisfactory clinical outcomes were achieved (Table 3).

**Table 3:** Postoperative data [case (%)/(x¯ ± s)].

Postoperative data	Data
The time of respirator (h)	40.95 ± 16.41
Length of ICU (day)	7 ± 3.97
Length of stay (day)	17.83 ± 5.27

## Discussion

Acute type A aortic dissection represents one of the most critical conditions within acute aortic syndrome. The procedure developed by Professor Sun Lizhong, known as Sun's procedure, is tailored to address the unique characteristics of aortic dissection prevalent in our country. Building upon the “frozen elephant trunk technique”, Professor Sun developed an approach that integrates aortic arch replacement with stented elephant trunk placement. this technique effectively eliminates aortic arch dissection, significantly improve false lumen occlusion rate in descending aortic dissection, and reduces or even negates the need for secondary surgeries of the descending thoracic aorta (3). The aortic arch comprises three important branches: the innominate artery, the left common carotid artery, and the LSA. Due to anatomical complexities such as obesity, dysplasia, or the enlargement of the false lumen in a dissecting aneurysm, these branches can be challenging to expose. This difficulty is exacerbated by the posterior displacement of the brachiocephalic vessels, which can lead complications such as recurrent laryngeal and phrenic nerve injury, anastomotic bleeding, stump bleeding, and distal recurrent bleeding (6). In addition,this difficulty prolong the CPB and HCA times, which can activate renal failure (7). Consequently, vascular reconstruction of the aortic arch in acute type A aortic dissection has long been a focal point in aortic surgery.

Current methods for aortic arch vascular reconstruction include: (1) The classic Sun's procedure(total aortic arch replacement with frozen elephant trunk technique). This technique uses four-branch vessels to reconstruct the aortic arch, effectively eliminating dissection and providing accurate anastomosis with favorable short- and long-term outcomes. A portion of the distal end of the stented elephant trunk was reserved as an anastomotic margin to provide sufficient vascular material for future combined replacement of the thoracic and abdominal aorta to anastomosis with the new stent or artificial blood vessel.Therefore, All patients underwent Sun's procedure.

However, it is associated with extended operative times, significant trauma, anastomotis, and a steep learning curve. The complexity of the procedure and the risk of massive bleeding in emergency situations, especially when vascular tissue is fragile, pose substantial challenges (8). (2) The island flap anastomosis. This method, as described by Fang Ran, reduces the number of anastomose, simplifies the surgical procedure, and shortens the duration of hypothermic circulatory arrest. However, it does not entirely eradicate the interlayer of the aortic arch, which may leave pathological vascular tissue. This approach can lead to difficulties in managing anastomotic bleeding if it occurs, as visualization and control can be extremely challenging (9). (3) Thoracic endovascular fenestration(TEF)with a hybrid prothesis. Combining surgical and interventional methods, TEF with hybrid prothesis aim to reduce operational complexity and simplify the procedure while addressing aortic arch pathologies. Despite their advantages, hybrid techniques face challenges such as specific requirements for stent anchoring, variable ranges of interlayer involvement, and potential complications such as persistent interlining and endoleaks. These techniques demand advanced skills, extensive learning curves, and substantial costs.

The treatment methods of LSA stump in classic Sun's surgery arch reconstruction are as follows: (1) Silk ligation method. This technique involves litigating the LSA stump with one or two silk threads. Due to the deep anatomical location of the LSA, this method is challenging and often involves operating in a region with less healthy arterial tissue. Consequently, if residual bleeding occurs, it can be difficult to control and may lead to severe complications. Additionally, residual aneurysm expansion may require secondary surgeries (10). (2) Sliding suture closure. In this method, the LSA stump is closed using a 5–0 prolene sliding suture. Although this method partially reduces the difficulty associated with deep tissue and fragile structures compared to silk litigation, it still presents issues such as difficulty in suturing, potential stump bleeding, and long-term aneurysmal expansion. (3) Innominate and Left Common Carotid Artery Reconstruction Only. In patients with a deeply located LSA but adequate collateral circulation from the circle of Willis and the left vertebral artery, this method reconstructs the blood supply of the LSA by anastomosing one of the four branch vessels to LSA through the left thoracic cavity (11). However, this approach may still encounter short-term stump bleeding and insufficient blood supply to the brain and left upper limb, particularly if vascular disease affects the innominate artery, unilateral vertebral artery or circle of Willis in the long term (12). (4) Trunk “window” stent technology. This method involves placing a stent at the position of the LSA window to reduce LSA operation complexity and improve the vascular anastomosis plane (13). However, it can increase the duration of circulatory arrest, raising the risk of lower body ischemia (14). Additionally, it is not suitable for cases with extensive LSA interlayer involvement and may face issues such as stent displacement, colostomy-related internal leakage, and suboptimal stent performance due to the distance between the LSA openings. An example is the Jin Yuan Qi risk (15).

Inspired by the endoscopic application of Hem-o-lok vascular clips for sealing vascular stumps (16), we explored their application for LSA stump management. Hem-o-lok vascular clips are made from multi-polymer inert plastic with a tough texture, are antibacterial, and do not elicit tissue rejection or cause cutting effects on vascular tissue. Moreover, the inner layer of the clips has anti-slip teeth and a locking mechanism that enhances stability and accuracy in clamping. They also allow for residual activity of the vascular ligation (17). The long-handled design of the Hem-o-lok clips facilitates rapid and safe ligation, reducing surgical time. We applied Hem-o-lok clips to manage the LSA stump in our procedures to minimize complications associated with deep exposure and suturing difficulties. The use of Hem-o-lok clips has shown advantages in avoiding bleeding and preventing aneurysmal expansion. Additionally, these clips do not create artifacts on x-ray, MRI, or CT scans, allowing for clear postoperative imaging and assessment (18).

A total of 38 patients were treated using two Hem-o-lok clips to occlude the LSA stump (Figure 1), with the preoperative LSA diameter measured at 10.98 ± 1.24 mm by CTA. However, it was observed that two side-by-side Hem-o-lok clips occupied approximately 1.5–2.0 cm of the LSA length, which made it difficult to anastomat the distal LSA with the artificial branch vessel. Therefore, this method was deemed unsuitable for patients with insufficient LSA length. To address the issue, our center modified the method by first suturing the stump with a 5–0 prolene sliding suture and then reinforcing the LSA stump with a single Hem-o-lok clip. This modification retained the advantages of Hem-o-lok clips while effectively addressing the problem of excessive length occupation by side-by-side clips. Based on our experience, we summarized the following application techniques: (1) Resistance. During deep hypothermic circulatory arrest after fully freeing the LSA, use a vascular clamp to occlude the LSA root. An assistant may be necessary to expose the LSA adequately by pulling down the clamp to assist with root occlusion. (2) Margin. Leave a margin of 3–5 mm from the broken LSA (for suturing) while placing the blocking clamp. (3) Suturing. Close the LSA stump with continuous 5–0 prolene sutures, then release the clamp and secure the knot. (4) Clamping. Use non-damaging forceps to pull the LSA stump, ensuring it protrudes from the LSA root. Apply the Hem-o-lok clip completely around the LSA root with the lock side embedded in the tissue, confirming a secure lock upon clicking (19) (Figure 2). Using this method, 54 patients were treated with the 5–0 prolene sliding suture followed by a single Hem-o-lok clip for LSA stump management. The preoperative LSA diameter was measured at 11.63 ± 1.66 mm by CTA. Since Hem-o-lok clips are suitable for vessels with diameters ranging from 3 to 16 mm, this method was not applied to patients with an LSA diameter greater than 15 mm. For four patients, the LSA stump was closed using only the 5–0 prolene sliding suture, with a preoperative LSA diameter measured at 14.64 ± 0.71 mm. In cases where the vertebral artery is deeper and poses exposure and anastomosis challenges, Hem-o-lok clamping can also be utilized for vertebral artery stump closure, reducing the complexity of deep position operations and saving operative time.

## Conclusion

To conclude, We could demonstrate that the simplified approach of Hem-o-lok closure of the LSA stump largely mitigates the difficulty in the LSA operation process,shortens the time of operation and reduces bleeding risk, thus effectively improving a patient's prognosis and yielding satisfactory clinical outcomes. The limitation of our study is that the indications for this surgical approach is not applied to patients with an LSA diameter greater than 15 mm.Short follow-up period and limited case numbers present certain limitations, necessitating further long-term follow-up to assess the sustained clinical efficacy of this technique.

## Data Availability

The original contributions presented in the study are included in the article/Supplementary Material, further inquiries can be directed to the corresponding author.

## References

[1] Liu ZG Sun LZ Chang Q Zhu JM Dong C Yu CT Should the "elephant trunk" be skeletonized? Total arch replacement combined with stented elephant trunk implantation for Stanford type A aortic dissection. J Thorac Cardiovasc Surg. (2006) 131(1):107–13. 10.1016/j.jtcvs.2005.09.015 16399301

[2] Khan IA Nair CK. Clinical, diagnostic, and management perspectives of aortic dissection. Chest. (2002) 122(1):311–28. 10.1378/chest.122.1.311 12114376

[3] Ma WG Zheng J Dong SB Lu W Sun K Qi RD Sun’s procedure of total arch replacement using a tetrafurcated graft with stented elephant trunk implantation: analysis of early outcome in 398 patients with acute type A aortic dissection. Ann Cardiothorac Surg. (2013) 2(5):621–8. 10.3978/j.issn.2225-319X.2013.09.06 24109570 PMC3791189

[4] Bekkers JA Raap GB Takkenberg JJ Bogers AJ. Acute type A aortic dissection: long-term results and reoperations. Eur J Cardiothorac Surg. (2013) 43(2):389–96. 10.1093/ejcts/ezs342 22677353

[5] Pagni S Ganzel BL Trivedi JR Singh R Mascio CE Austin EH Early and midterm outcomes following surgery for acute type A aortic dissection. J Card Surg. (2013) 28(5):543–9. 10.1111/jocs.12170 23909254

[6] Kandil E Abdel Khalek M Aslam R Friedlander P Bellows CF Slakey D. Recurrent laryngeal nerve: significance of the anterior extralaryngeal branch. Surgery. (2011) 149(6):820–4. 10.1016/j.surg.2011.02.012 21497872

[7] Sansone F Morgante A Ceresa F Salamone G Patanè F. Prognostic implications of acute renal failure after surgery for type A acute aortic dissection. Aorta. (2015) 3(3):91–7. 10.12945/j.aorta.2015.14.022 27069938 PMC4820344

[8] Erbel R Aboyans V Boileau C Bossone E Bartolomeo RD Eggebrecht H 2014 ESC guidelines on the diagnosis and treatment of aortic diseases: document covering acute and chronic aortic diseases of the thoracic and abdominal aorta of the adult. The task force for the diagnosis and treatment of aortic diseases of the European Society of Cardiology (ESC). Eur Heart J. (2014) 35(41):2873–926. 10.1093/eurheartj/ehu281. Erratum in: *Eur Heart J*. (2015) **36**(41):2779. doi: 10.1093/eurheartj/ehv178. 25173340

[9] Qin J Zhao Z Wang R Ye K Li W Liu X *In situ* Laser fenestration is a feasible method for revascularization of aortic arch during thoracic endovascular aortic repair. J Am Heart Assoc. (2017) 6(4):e004542. 10.1161/JAHA.116.004542 28432073 PMC5532990

[10] Lin FY Tseng YH Huang JW Hsieh CC Chen HM Chiu CC Fate of distal aorta after acute type A aortic dissection repair: change and persistency of postoperative false lumen status. Int J Cardiol. (2018) 266:50–5. 10.1016/j.ijcard.2018.01.010 29887472

[11] Goebel N Holder SA Huether F Bail DHL Franke UFW. Left subclavian artery sacrifice in acute aortic dissection repair using the frozen elephant trunk. Thorac Cardiovasc Surg. (2022) 70(8):623–9. 10.1055/s-0041-1741058 35038756

[12] Toda K Taniguchi K Masai T Takahashi T Kuki S Sawa Y Arch aneurysm repair with long elephant trunk: a 10-year experience in 111 patients. Ann Thorac Surg. (2009) 88(1):16–22. 10.1016/j.athoracsur.2009.03.092 19559181

[13] Tang Y Liao Z Han L Xu Z. Left subclavian artery fenestration: a novel treatment strategy for acute type A aortic dissection. Ann Thorac Surg. (2016) 101(1):95–9. 10.1016/j.athoracsur.2015.06.069 26347120

[14] Chiu P Goldstone AB Schaffer JM Lingala B Miller DC Mitchell RS Endovascular versus open repair of intact descending thoracic aortic aneurysms. J Am Coll Cardiol. (2019) 73(6):643–51. 10.1016/j.jacc.2018.10.086 30765029 PMC6675458

[15] Chen LW Dai XF Lu L Zhang GC Cao H. Extensive primary repair of the thoracic aorta in acute type a aortic dissection by means of ascending aorta replacement combined with open placement of triple-branched stent graft: early results. Circulation. (2010) 122(14):1373–8. 10.1161/CIRCULATIONAHA.110.946012 20855660

[16] Roh YJ Kim JW Jeon TJ Park JY. Common bile duct stone development due to a Hem-o-lok clip migration: a rare complication of laparoscopic cholecystectomy. BMJ Case Rep. (2019) 12(7):e230178. 10.1136/bcr-2019-230178 31352393 PMC6663218

[17] Verhoeven EL Katsargyris A Bekkema F Oikonomou K Zeebregts CJ Ritter W Editor’s choice—ten-year experience with endovascular repair of thoracoabdominal aortic aneurysms: results from 166 consecutive patients. Eur J Vasc Endovasc Surg. (2015) 49(5):524–31. 10.1016/j.ejvs.2014.11.018 25599593

[18] Faure EM Canaud L Marty-Ané C Alric P. Hybrid aortic arch repair for dissecting aneurysm. J Thorac Cardiovasc Surg. (2016) 152(1):162–8. 10.1016/j.jtcvs.2016.03.020 27068438

[19] Chou HW Chan CY Chang CH Lin CF Chen YS Wang SS Comparisons of aortic remodelling and outcomes after endovascular repair of acute and chronic complicated type B aortic dissections. Interact Cardiovasc Thorac Surg. (2018) 27(5):733–41. 10.1093/icvts/ivy167 29796637

